# New insights into irritable bowel syndrome pathophysiological mechanisms: contribution of epigenetics

**DOI:** 10.1007/s00535-023-01997-6

**Published:** 2023-05-09

**Authors:** Giovanni Dothel, Maria Raffaella Barbaro, Aldo Di Vito, Gloria Ravegnini, Francesca Gorini, Sarah Monesmith, Emma Coschina, Eva Benuzzi, Daniele Fuschi, Marta Palombo, Francesca Bonomini, Fabiana Morroni, Patrizia Hrelia, Giovanni Barbara, Sabrina Angelini

**Affiliations:** 1 Department of Pharmacy and Biotechnology, University of Bologna, Bologna, Italy; 2 IRCCS Azienda Ospedaliero-Universitaria Di Bologna, Bologna, Italy; 3 Department of Medical and Surgical Sciences, University of Bologna, Bologna, Italy; 4 Inter-Departmental Center for Health Sciences & Technologies, CIRI-SDV, University of Bologna, Bologna, Italy; 5 Present Address: Connect By Circular Lab SRL, Madrid, Spain

**Keywords:** Irritable bowel syndrome, Epigenetic, DNA methylation, miRNA

## Abstract

Irritable bowel syndrome (IBS) is a complex multifactorial condition including alterations of the gut–brain axis, intestinal permeability, mucosal neuro-immune interactions, and microbiota imbalance. Recent advances proposed epigenetic factors as possible regulators of several mechanisms involved in IBS pathophysiology. These epigenetic factors include biomolecular mechanisms inducing chromosome-related and heritable changes in gene expression regardless of DNA coding sequence. Accordingly, altered gut microbiota may increase the production of metabolites such as sodium butyrate, a prominent inhibitor of histone deacetylases. Patients with IBS showed an increased amount of butyrate-producing microbial *phila* as well as an altered profile of methylated genes and micro-RNAs (miRNAs). Importantly, gene acetylation as well as specific miRNA profiles are involved in different IBS mechanisms and may be applied for future diagnostic purposes, especially to detect increased gut permeability and visceromotor dysfunctions. In this review, we summarize current knowledge of the role of epigenetics in IBS pathophysiology.

## Introduction

Irritable bowel syndrome (IBS) is a disorder of gut–brain interaction (DGBI) characterized by recurrent abdominal pain associated with defecation or change in bowel habits [1]. According to ROME IV criteria, four IBS subgroups are identified: IBS with diarrhoea (IBS-D), IBS with constipation (IBS-C), IBS with mixed bowel habits (IBS-M), and unclassified IBS (IBS-U) [2]. A large subgroup of IBS patients experiences extra-intestinal symptoms, including psychiatric and mood disorders [3–5]. Early life adverse events (EAEs), comprising psychological and physical stress as well as traumatic experiences during childhood have been identified as a predisposing factor for IBS development [6, 7]. Several factors are involved in the pathophysiology of IBS including unbalanced gut microbiota [8], low-grade immune activation [9], overactive serotonergic system [10], and intestinal barrier dysfunction [11]. Regarding immune system involvement, the observation of mucosal infiltration with immune cells, in particular mast cells, in IBS patients has been backed up by mechanistic evidence. This demonstrates an effect on the epithelial permeability, and enteric nervous system function [12–16]. More recently, epigenetic modifications such as chromatin remodelling, DNA methylation, and non-coding RNAs have been indicated among the players involved in IBS development (Tables 1 and 2) [17–21]. Additionally, gut microbiota can modulate the intestinal host’s gene expression by its metabolites and in turn can be epigenetically regulated by the host. Additionally, gut microbiota can modulate the intestinal host’s gene expression by its metabolites [22] and in turn can be epigenetically regulated by the host [23].

**Table 1:** Main ncRNAs involved in IBS pathophysiology and identified as regulators of IBS mechanisms

miRNA [ref]	miRNA expression (↓ or ↑) in IBS *vs* HC	Target gene or pathway (identified in human samples and/or cells)^#^	Observed correlation with GI function	Clinical condition and samples analysed (*n*); (human, cells, and animal model)
**Human studies**				
miR16 [112]	↓	5HT-4	Motility and stool form	IBS-D (*n* 14) & HC (*n* 17); Jejunum biopsies
miR-16a [19]	↓	CLDN2	Increased IP	IBS-D (*n* 43) & HC (*n* 23); Proximal jejunum biopsies (Watson capsule)
miR-24 [114]	↑	SERT	Increased VHS	IBS (*n* 10) & HC (*n* 10); intestinal mucosa biopsies
miR-29a				
[74]	↑	GLUL	Increased IP	IBS-D (*n* 19) & HC (*n* 10); duodenum and colon biopsies and blood microvesicles
[77]	↑	ZO-1 & CLDN1	Increased IP	IBS-D (*n* 21) & HC (*n* 16); colon biopsies
[118]	↑	HTR7	Increased VHA	IBS (*n* 10) & HC (*n* 10); colon biopsies
miR-29a/b [75]	↑	CLDN1 & NKRF	Increased IP	IBS-C (*n* 74), IBS-D (*n* 109), & HC (*n* 36); Colon and jejunal biopsies
miR-103 [112]	↓	5HT-4	Motility and stool form	IBS-D (*n* 14) & HC (*n* 17); Jejunum biopsies
miR-125b [19]	↓	CGN	Increased IP	IBS-D (*n* 43) & HC (*n* 23); Proximal jejunum biopsies (Watson capsule)
miR-148b-5p				
[82]	↑	RGS-2	Increased IP	IBS-D (*n* 20) & HC (*n* 20); HT-29 cells cultured with serum exosomes (IBS-exo & HC-exo)
[95]	↑	§		IBS-C (*n* 14), IBS-D (*n* 17), & HC (*n* 30);
miR-199a [18]	**↓**	TRPV1	Increased VHS	IBS-D (*n* 45) & HC (*n* 40); colon biopsies
miR-199b [66]	**↓**	/	Increased coliform count	IBS-C (*n* 20), IBS-D (*n* 18), IBS-M (*n* 32) & HC (*n* 20); serum
miR-219a-5p [81]	↓	ITGB1BP1, ABC transports C1 & C5, CAMK1D	Impaired barrier function	IBS-C (*n* 15), IBS-D (*n* 14), & HC (*n* 15); Colon biopsies
miR-338-3p [81]	↓	MAPK signaling	Increased VHS	IBS-C (*n* 15), IBS-D (*n* 14), & HC (*n* 15); Colon biopsies
miR-510 [111]	δ; functional variant 5HT3	Increased 5HT_3E_ expression	Symptoms associated with female IBS-D	IBS-C (*n* 100), IBS-D (*n* 200), & HC (*n* 100)—discovery study; IBS-D (*n* 119), & HC (*n* 195) – a replication study
lncRNA H19 [86]	↑	§z	Increased IP	IBS-D (*n* 10), & HC (*n* 10);
**Animal studies**				
miR-21-5p [65]	↑	PTEN & PDCD4	Increased IP	GF mice & Conventional mice
miR-24 [114]	↑	SERT	Increased VHS and inflammation	TNBS-induced IBS mouse model & Control
miR-29a				
[76]	↑	CLDN1, Collagen I, collagen IV, FBN & ITGB1,	Increased IP	IUGR PN (*n* 6) & NPN (*n* 12)
[77]	↑	ZO-1 & CLDN1	Increased IP	TNBS-induced IBS-D model & Control
[118]	↑	HTR7	Increased VHA	WAS-induced IBS models: miR-29a knockout & wild-type mice
miR-29/a/b [75]	↑	CLDN1 & NKRF	Increased IP	TNBS & WAS-induced IBS models: miR-29a/b knockout & wild-type mice
miR-144 [78]	↑	Occludin & ZO-1	Increased IP	Acid acetic-induced IBS-D rat model (*n* 20) & Control (*n* 20)
miR-181c-5p [101]	↑	IL1A, TNFα, IL2, IL6	Decreased low-grade inflammation	AWR-induced IBS rat model (*n* 56) & Control (*n* 15)
miR-200a [79]	↑	CNR1 & SERT	Increased VHA	Unpredictable chronic stress-induced IBS-D rat model (*n* 20) & Control (*n* 20)
miR-325-5p [141]	**↓**	CCL2	Increased VHS	Acid acetic rat model & CRD used to induce pain behavior
miR-495 [140]	**↓**	PI3K/AKT path via PKIB	Increased VHS	Acid acetic-induced IBS-D mouse model (*n* 10) & Control (*n* 10)
**In vitro studies**				
miR200b [80]	↑	TNFα	Decreased TEER & paracellular permeability	Caco2 cell line
miR-490-5p [91]	↑	Tryptase & PAR2	Promotes cell proliferation and resistance to apoptosis	p815 mast cell line

^**#**^Target gene expression inversely correlated with miRNAs expression; ^**§**^Target not investigated; ^δ^No change in miRNA expression but disruption of an miRNA-binding site;

*AWR* abdominal withdraw reflection, *CAMK1D* calcium/calmodulin dependent protein kinase ID, *CCL2* C–C motif chemokine ligand 2, *CLDN1* Claudin 1, *CLDN2* Claudin 2, *CGN* Cingulin, *CRD* colorectal distention, *FBN* fibronectin, *IP* intestinal permeability, *ITGB1* Integrin-β1, *ITGB1BP1* integrin subunit beta 1 binding protein 1, *IUGR PN* intrauterine growth restricted porcine neonates, *NPN* normal porcine neonates, *RGS-2* regulator of G protein signaling-2, *TEER* transepithelial electrical resistance *TNBS* 2,4,6- trinitrobenzenesulfonic, *VHA* visceral hyperalgesia, *VHS* visceral hypersensitivity, *WAS* water avoidance stress

**Table 2:** Gene methylations involved in IBS mechanisms

Methylated Genes	Expression (↓ or ↑)	Regulation &/or observed function	Biomarkers analysed	Clinical condition and samples analysed (*n*); (human, cells and animal model); biological sources	Reference
**Human studies**					
*SSPO, GSTM1, GSTM5, TPPP*	↑	Association of *SSPO* methylation with high HADs and PSS scores	HAD and PSS scores	IBS-D (*n* 10), IBS-C (*n* 8), IBS-M (*n* 9), HC (*n* 23); whole blood (PBMCs)	[20]
*ADCYAP1*	↓				
AKAP12; PRKAR1B	↑				
**Animal studies**					
*GR, CRF*	GR↑, CRF↓	Increase visceral pain	VMR to CRD; GR, CFR expression	WAS rat model & HC; amygdala tissue	[137]
H3K9 methylation in the promoter region of claudin-1, ZOs, and occludin	↑	IL-6 promotes H3K9me2/me3, preventing GR transcriptional factors binding on the TJs genes promoter region; with the consequent increase in permeability and visceral pain	IL-6, GR, claudin-1, ZOs, occludin	WAS rat model & HC	[71]
*GR (NC3R1), CNR1*	↑	WAS increased DNMT1-mediated CNR1 methylation	VMR to CRD, GR, TRPV1, CNR1	WAS rat model & HC	[21]

*CNR1* cannabinoid receptor 1, *COX2* cyclooxygenase-2, *CRD* colorectal distension, *CRF* corticotropin-releasing factor, *DNMT1* DNA methyltransferases 1, *GR* glucocorticoid receptor, *HC* healthy control, *IFNγ* interferon γ, *IL-1β* interleukin-1β *IL-6* interleukin-6, *IL-8* interleukin-8, *IkB* inhibitor of kB, *NF-κB* nuclear factor kappa-light-chain-enhancer of activated B cells, *PBMCs* peripheral blood mononuclear cells, *PSS* perceived stress scale, *VMR* visceromotor response, *WAS* water-avoidance stress

In this review, we will focus on current findings about epigenetic mechanisms and non-coding RNA identified in IBS patients and in early life stress animal models, thus revealing possible novel targets for future diagnostic and therapeutic tools.

## Epigenetic and non-coding RNAs

The term “epigenetics” refers to the whole biomolecular mechanism inducing chromosome-related, heritable changes of gene expression, regardless of DNA coding sequence [24]. Hence, the current definition comprises upstream modifications of chromatin and DNA structure, occurring at the gene promoter site but not the downstream modulation of transcripts provided by non-coding RNA molecules. However, for convenience, in this review the locution “epigenetic modification/regulation/factors” will also include non-coding RNA functions.

**Fig. 1:**
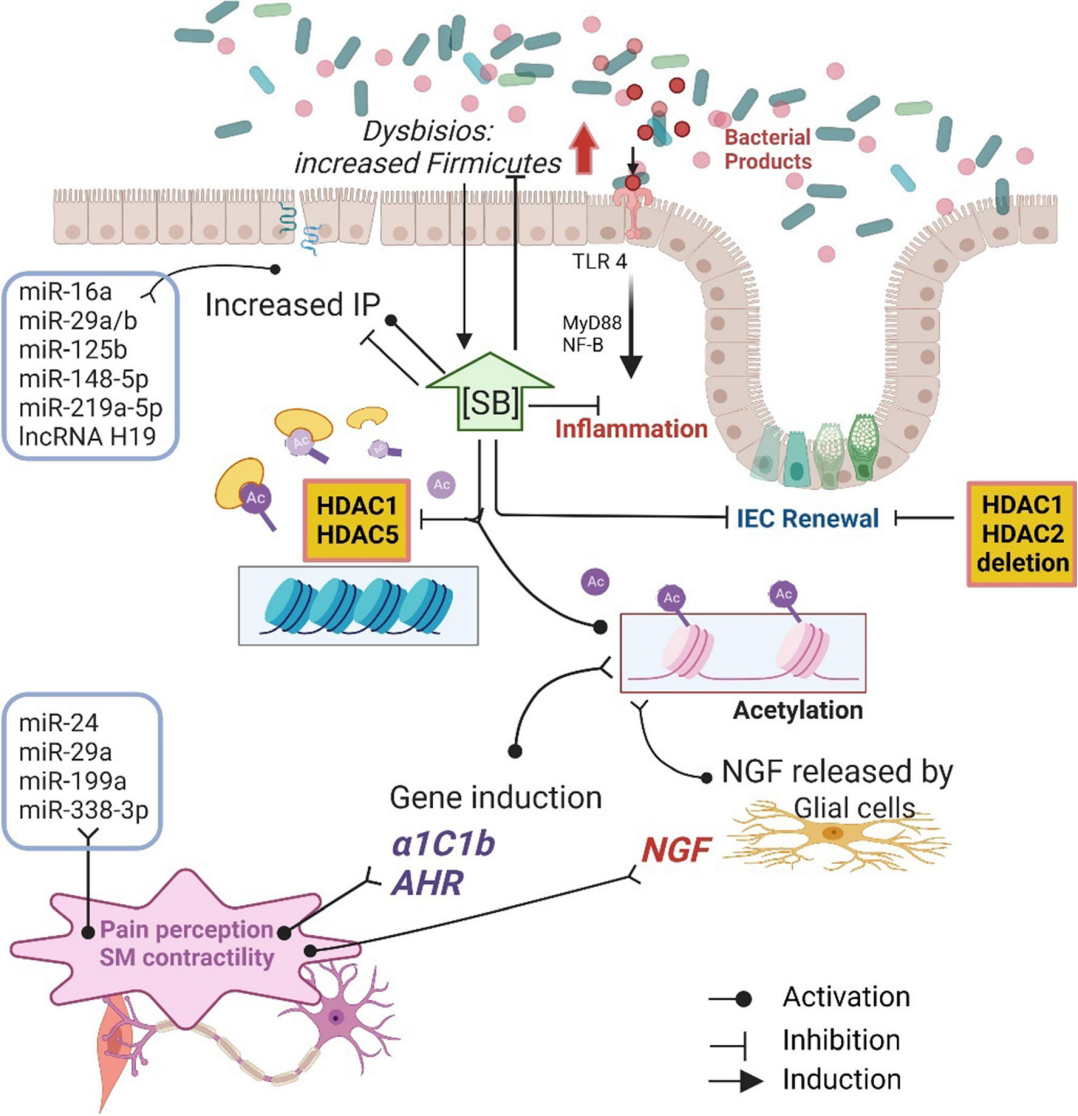
Epigenetic factors involved in gut–brain axis dysregulation – Histone acetylation and miRNA expression triggering biomolecular pathways involved in IBS. The underlying hypothesis envisages miRNA such as miR-24 and miR-29a having a direct involvement in pain perception and increased IP. An akin effect is induced by SB, a great HDAC inhibitor, produced at high levels by *Firmicutes.* In contrast, high SB levels were shown to restore IP and dysbiosis. Increased histone acetylation elicits NGF production by enteric glial cells resulting in higher SM contractility and pain perception. High SB levels reduce TLR4-mediated inflammation and, allegedly by a dose-dependent mechanism, both impair and restore intestinal permeability. *AHR* aromatic hydrocarbon receptor, *IEC* intestinal epithelial cells, *NGF* nerve growth factor, *SB* sodium butyrate, *VIP* vasoactive intestinal protein

Nucleosomes represent chromatin’s functional units and determine its relaxed or condensed form, namely euchromatin and heterochromatin, respectively. Nucleosomes are octamers composed of 4 couples of histones with the genomic DNA wrapped around each one. Histone acetylation induces histone distancing and a loosened chromatin structure, favouring the access of transcription complexes to the promoter sequence. Conversely, the loss of acetylic groups condenses chromatin structure and inhibits gene transcription (Fig. 1) [25]. Histone acetyl transferase (HATs) and histone deacetylases (HDACs) are the enzymes responsible for the association and detachment of acetylic groups on histones. In addition, histones can undergo several other enzyme-mediated modifications such as methylation, biotinylation, phosphorylation, sumoylation, ubiquitination, and serotonylation (see following paragraph), which is more recently discovered. These processes may either promote or repress gene transcription [26]. Conversely, methylation of genomic DNA on the 5’ terminal in correspondence to cytosine–guanine dinucleotides inhibits the attachment of transcription complexes. DNA methyl transferase (DNMTs) and ten-eleven translocation enzymes mediate cytosine methylation and demethylation, respectively [27].

Around 90% of the total RNA in a normal cell do not translate genetic information into any protein product. A dramatic step-forward of last decade clarified that this remarkable component of the transcriptome owes regulatory functions. Nowadays, research is focused on the causal links between non-coding RNAs and a vast number of biological functions in health and disease [28, 29]. Among the various categories [30], much attention has been shed on microRNA (miRNA) and long non-coding RNA (lncRNA) and their involvement in inflammatory and functional GI disorders [31, 32]. miRNAs are 20–22-base-long RNA oligomers modulating gene expression by binding complementary mRNAs on specific sequences and inducing the inhibition of the translation or the degradation of mRNA [33, 34]. Differently, lncRNAs are oligo sequences spanning 200–100.000 nucleotides in length, stemming from DNA regions flanking gene promoter sequences. lncRNA can transcribe adjunctive miRNA to form RNA macromolecules with the capacity to absorb target miRNA through a sponge-like mechanism. The function of lncRNAs has not yet been elucidated, but they likely have several regulatory roles, including the modulation of gene expression by chromatin remodelling [35]. Moreover, non-coding RNAs can signal outside the cellular space complexed with RNA-binding proteins or included in extracellular vesicles [36, 37].

## Epigenetic and non-coding RNAs in IBS

### Host–microbiome interaction

Perturbations of the complex homeostasis between host and intestinal microbial species in IBS have been largely studied in the last decade. Luminal bacteria participate in digestive functions including the digestion of Fermentable, oligo-, di-, monosaccharides, and polyols (FODMAPs). These are the primary source of short-chain fatty acids (SCFAs) which are the key factors in the interplay with the host [17, 38–40] and are likely involved in IBS pathophysiology. Available evidence suggests a role for SCFAs in intestinal inflammation, intestinal barrier integrity, motility, and gut–brain axis regulation [41]. In particular, among the most abundant SCFAs in the colon, Tana and colleagues demonstrated that acetate and propionate are increased, together with total organic acids, in IBS patients compared to controls, and positively correlated with symptom severity [42]. Another SCFA, Sodium butyrate (SB), a renowned HDAC inhibitor [43, 44] which has gained much attention for its debated role as an immune modulator [38, 45–47], it is currently studied predominantly in IBS pathophysiology [40]. SB exerts its activity by either receptor coupling or by the activation of specific genes involved in intestinal motility and immune activation [45, 48, 49]. Recent studies unravelled a pleiotropic effect of SB with more than 2400 genes regulated by histone acetylation as a result of SB-induced HDAC inhibition (Fig. 1, Table 3). Although there are still no data on the involvement of the SB-HDAC mechanism in IBS, it is reasonable to assume it on the basis of the following evidence. Additionally, SB exposure strongly affects the expression of ligand-activated aromatic hydrocarbon receptor (AHR) in cell lines [50], which in turn mediates stem cell proliferation as well several other processes including xenobiotic metabolism, adaptive immunity and, as more recently assessed, intestinal motility [51–53]. Indeed, Obata and colleagues elegantly detailed a causal link between microbiota colonization and colonic motor function by the activity of AHR [53]. Microbiota colonization of germ-free (GF) mice induced AHR expression in colonic neurons, which in turn activated different genes, including the *kcnj12*, coding for the Kir2.2 inward rectifier potassium channel, involved in neuronal depolarization. In addition, AHR knock-out mice showed a faster intestinal transit compared to wild-type mice as well as a lower amplitude of colonic migrating motor complex of isolated colonic muscular strips. This indicates that AHR increases colon peristalsis by an autonomous fashion regardless of central circuits [53]. Furthermore, AHR promoter activity was increased by 5 to 7 folds in different cell lines upon HDAC inhibition and in particular, because of n-butyrate treatment [50]. In addition, Marinelli and colleagues indicated SB as a possible AHR ligand [49]. In line with these studies, colonization of GF mice with microbiota derived by conventionally fed mice increased histone 3 (H3) and histone 4 (H4) acetylation which correlated with SCFA amount [54]. Analogously, Gills and colleagues described an increased acetylation of H3 and H4 localized at the promoter region of the serotonin transporter (SERT). A lower SERT level was detected in response to SB treatment in vitro as well as in vivo in the distal ileum and colon tract of mice fed with pectin, a soluble dietary fibre producing elevated levels of SCFA (see paragraph on serotonin signalling) [55]. Finally, a further alteration of luminal content could be exerted by high SB by altering the homeostasis of sodium and calcium transport [56]. Accordingly, a recent meta-analysis suggests an ameliorative effect of a low-FODMAP diet on IBS symptoms [57]. Moreover, luminal *Firmicutes*, major contributors of SB production in the human gut, were increased in patients with IBS [58], as well as in animal models, [59]; their intestinal load also positively correlated with IBS symptom scores [60]. Although it is clear that SB is involved in key pathways of IBS pathophysiology, further studies are necessary to clarify its role, particularly in relation to other factors influencing its concentration. Indeed, experimental data demonstrated that the effect of SB is dose-dependent, therefore, it is fundamental to consider factors including diet, antibiotics, and probiotics, which could influence its final concentration [61].

**Table 3:** Histone acetylation associated with IBS mechanisms

Histone ID acetylation site	Acetylation (↑ or↓)	Regulation &/or observed function	Clinical condition and samples analysed (*n*); (human, cells, and animal model); biological sources	Reference
**Animal studies**				
H4K12	↓	HDAC inhibition ameliorate VHS	Stress-induced VHS rat model (maternal separation stress) (n 20) & HC (n 10); spinal and lumbosacral DRGs	[128]
H3K9—*CACNA1*	↑	increased intestinal motility associated with VIP and α1C1b increased expression	TNBS rat model (*n* 6) & HC (*n* 6); colon resection	[124]
Overall acetylation	↑	SB induces NGF released by EGCs	SB supplemented CRL2690 cells; Colonic hypersensitivity induced by butyrate enemas in rats (n 8 treated & n 8 control); Colon resections	[125]
Overall acetylation	↓	HDAC inhibition ameliorates VHS	WAS rat model & HC; amygdala tissue	[137]
H3	↑	BDNF-mediated VHS	CPS and chronic stress in adult offspring (WAS or FSS); spinal and lumbosacral DRGs	[133]
HDAC1	↓			
H3K9, H3K18	↓	HDAC inhibition ameliorate VHS	FSS rat model & HC	[138]
H3K9	↓	HDAC inhibition ameliorates estradiol-induced VHS	ovariectomized rats, 17β-estradiol treated & untreated	[139]
	↑	Increased VHS; HDAC inhibition ameliorate VHS via SERT expression	Unpredictable early life stress rat model; central nucleus of the amygdala	[136]
H3	↑	Increased VHS via TRPV1 overexpression	WAS rat model; spinal and lumbosacral DRGs	[21]
**In vitro studies**				
Overall acetylation	↓	SCFAs reduce LPS-induced permeability, inhibiting NLPR3 and autophagy	SCFAs treated Caco-2 cell line	[38]
Overall acetylation	↓	SB induces IL-10RA by STAT3 activation resulting in enhanced permeability	SB treated T84 cell line	[17]
H3, H4	↑	HDAC inhibitions decreased SERT expression	SB or TSA-treated Caco-2 cell line	[55]

Specific sites of acetylation are labelled with a number of acetylated histones followed by the number of the acetylated lysine on the histone tail where stated (e.g. H3K9)

*BDNF* brain-derived neuronal growth factor, *CPS* chronic prenatal stress, *FSS* forced swim stress, *TNBS* tri-nitro benzene sulphonic acid, *TSA* trichostatin, *WAS* water avoidance stress, *HDAC* histone deacetylases, *DRGs* dorsal root ganglia, *MS* maternal separation, *SAHA* suberoylanilide hydroxamic acid, *VIP* vasoactive intestinal peptide, *SB* sodium butyrate, *NGF* neuronal growth factor, *EGCs* enteric glial cells, *VMR* visceromotor response, *CRD* colorectal distension, *SCFA* short chain fatty acids, *ZO-1* zonula occludens-1, *IL-1β* interleukin-1β, *IL-18* interleukin 18, *TSA* trichostatin A, *IL-10 RA* interleukin 10 receptor α, *WAS* water -avoidance-induced stress, *veh*. vehicle, *FS* forced swim, *DMSO* dimethyl sulphoxide, *mGluR* metabotropic glutamate receptor, *CRH* corticotropin-releasing hormone, *GR* glucocorticoid receptor, *CeA* central nucleus of the amygdala, *HAT* histone acetyl-transferases, *TRPV1* transient receptor potential cation channel subfamily V member 1

A recent study investigated the effects of SB on visceral sensitivity, highlighting the role of Interleukin-1 Receptor-Associated Kinase 1 (IRAK1). Despite the reduced sample size involved in the study, results showed the SB ability to reduce IRAK1 expression both in vivo and in vitro and, in turn, to smooth the visceral hypersensitivity in the IBS mice model [62].

Intestinal miRNAs act as modulators at host-microbial interface. Exosomes containing specific miRNA sequences derived by intestinal epithelial cells (IECs) were detected in mice feces. In addition, transgenic mice unable to produce IECs-derived miRNA showed an increased luminal content of *Firmicutes* and *Proteobacteria phila*, as well as variations of specific bacterial families [23]. Moreover, chemical-induced inflammation altered the miRNA profile in mouse models, further confirming the importance of IEC as a gut primary source of miRNA [63].

Several studies showed a distinct miRNA pattern in GF mice compared to mice with conventional gut microbiota [59, 64] (Fig. 1 and Table 1). In addition, miRNA profiling comparing GF and antibiotic-treated mice indicated specific downregulated miRNA, strengthening the hypothesis of an eventual application of miRNA patterns as biomarkers for microbiota impairment and dysbiosis [59, 65]. In line with this, a lower level of miR-199b was detected in blood samples of patients with IBS compared to healthy controls (HC), which inversely correlated with a higher concentration of coliform bacteria [66]. miR-199b is encoded within the introns of the *DNM1* gene, a member of the dynamin GTPase family proteins, crucial in the formation of endocytic vesicles, a fundamental step in endocytosis [67]. A study by Zhou and co-workers on IBs patients correlated a decreased miR-199b expression with augmented visceral hypersensitivity and abdominal pain through TRPV1 upregulation [18]. Additionally, considering the role of miR-199a/b in endocytosis, its involvement in intestinal barrier permeability cannot be excluded. A further study on GF mice colonized with faecal content, derived from naïve mice, showed reduced expression of miR-665, targeting Abcc3, which is a transport protein involved in biliary and intestinal excretion of organic anions [68]. Overall, in this study, miRNA concentration was found to increase in the colon compared to ileum tissue. This finding could reflect a positive correlation of miRNA content with increasing bacterial load [68]. Interestingly, there was a higher level of miRNA in the lower intestinal tract of both GF and conventional mice compared to the small intestine. The authors ascribed this difference to different structures and functions of the small and large intestines. In this regard, it would be interesting to address whether the intestinal microbiota might have contributed phylogenetically to the inherited genetic traits also through miRNA induction. Finally, other microbial species such as virus and fungi showed the capacity to influence host immune defences through the inter-kingdom transfer of miRNA or miRNA-like molecules [69]. In this context, it is worth mentioning that several viral species could influence bacterial homeostasis through miRNA expression [70]. However, the impact of this interaction in shaping the host’s gene expression is still undetermined.

### Intestinal barrier permeability

The intestinal barrier is the first interface between the gastrointestinal tract and the environment. It is essential to avoid the passage of harmful antigens and bacteria into the deeper layers of the gut wall, but on the other side, it must allow the passage of nutrients, ions, and molecules useful for the body [11].

A recent study by Wiley and coworkers demonstrated the IL-6 ability to promote the methylation of lysine 9 on histone 3 (H3K9me2/me3) and prevent the binding of transcriptional factors, like glucocorticoid receptor (GR), in the promoter region of tight junction (TJ) genes in young adult male rats subjected to water avoidance and human cells [71]. In addition, intestinal epithelial HDAC1/2 deficiency decreases the expression of claudin-3 (CLDN3), a component of epithelial thigh junctions [72]. In line with this, local ablation of HDAC1 and HDAC2 in the intestine of mouse models and in cultured intestinal organoids induced a marked loss of enteric stem cells, which could accordingly impair epithelial renewal and barrier integrity [73]. Furthermore, lysine 9 on histone 3 (H3K9) methylation decreases claudin-1 (CLDN1), ZOs, and occludin expression in epithelial cells, resulting in paracellular permeability increase [71].

An altered miRNA profile has been related to intestinal barrier dysfunctions in IBS-D patients [19, 74–77]. In particular, a study reported increased miR-29a expression in gut tissues and blood microvesicles derived from patients with IBS, showing higher lactulose/mannitol urine fraction [i.e., a measure of intestinal permeability (IP)] compared to HC [74]. In the same study, the authors highlighted significantly enhanced epithelial permeability following miR-29a overexpression, whereas an opposite effect was observed after miR-29a inhibition in human colonic epithelial cells (FHC) and human IECs of the small intestine (FHs74Int) [74]. A further study identified the miRNA family members miR-29a and miR-29b associated with increased IP in a subset of patients with IBS-D characterized by increased IP, but not in IBS-D patients, showing normal permeability, in IBS-C patients, or in HC [75]. In particular, in this study higher miR-29a/b levels were associated with a lower expression of tight junction components and with the disruption of CLDN1 and nuclear factor repressing -κb- factor (NKRF) mRNAs. Furthermore, the authors showed a milder increase of IP in miR-29a/b^−/−^ mice subjected to either water avoidance stress (WAS) or trinitrobenzene sulfonic acid (TNBS) treatment, compared to wild-type mice [75]. In line with these findings, increased miR-29a level was associated with higher IP in a model of intrauterine growth restriction foetus in pigs [76]. Interestingly, the increase in miR-29a was associated with a decreased expression of TJs and extracellular matrix proteins, which impair cell growth and intestinal epithelial integrity [76] (Fig. 1). These findings are supported by an in vitro experiment showing an improvement in the monolayer integrity by increasing cell proliferation and transepithelial electric resistance following miR-29a inhibition [76]. More recently, a study reported that miRNA-29a was upregulated in the colonic epithelium of patients with IBS-D, while ZO-1 and CLDN1 were downregulated and the apical junctional complex was discontinuous [77]. In the same study, a TNBS-induced IBS-D mouse model, the treatment with miRNa-29a inhibitor induced an increase of ZO-1 and CLDN1 expression, confirming their role in the intestinal mucosal barrier [77]. Other miRNAs potentially involved in IP impairments are miR-21-5p, miR-144, and miR-200a—identified in animal models [65, 78, 79]—and miR-16 and miR-125b-5p—identified in humans [19]. Regarding animal models, Nakata and colleagues described that miR-21-5p induces ADP ribosylation factor 4 (ARF4), a member of the Ras GTPase superfamily which impairs intestinal barrier functions, via silencing ARF4-negative regulators PTEN and PDCD4 [65]. Another study highlighted that higher expression of miR-144, targeting ZO-1 and occludin mRNAs was associated with increased IP in a mouse model of IBS-D [78]. Regarding the miR-200 family, an in vitro study demonstrated that miR-200b expression inhibits paracellular permeability hindering TNFα-induced disruption of TJs [80]. Interestingly, upregulation of a member of the same family, miR-200a, increased visceromotor response in vivo by targeting cannabinoid receptor 1 (CNR1) and serotonin transporter (SERT) transcripts [79]. On the contrary, decreased miR-16 and miR-125b-5p levels correlated with increased IP in colonic biopsies of IBS-D patients compared to HC [19]. In particular, authors demonstrated these two miRNAs regulate the expression of TJ proteins encoded by CLDN2 (target of miR-16) and CNG (target of miR-125b) which, in turn, modulate intestinal epithelial barrier function and correlate with major clinical symptoms [19]. A recent work evaluated miRNA levels in the sigmoid biopsies of IBS patients and identified two miRNA, miR-219a-5p and miR-338-3p, significantly decreased in IBS compared to controls [81]. In addition, miR-219a-5p inhibition induced a permeability increase in colonic epithelial cell line NCM460 while the inhibition of miR-338-3p caused alterations in the mitogen-activated protein kinase (MAPK) signalling [81]. A further study performed on human colonic epithelium HT-29 cells highlighted the involvement of miR-148b-5p in epithelial barrier regulation. In particular, HT-29 cells, incubated with serum-derived exosomes from patients with IBS, showed increased permeability by up-regulating miR-148b-5p to suppress RGS2 expression. Interestingly, the effect on permeability was abrogated by interfering with miR-148b-5p expression [82].

The impairment of intestinal permeability in IBS has also been associated with a decreased expression of aquaporins (AQP) particularly AQP 1,3 [83]. Interestingly, a further work showed a decreased expression of lncRNA H19, which positively correlated with AQP1 and AQP3, in IBS-D patients [84].

### Neuro-immune interactions

An increased number of mucosal immune cells, particularly mast cells, have been reported in IBS patients, and low-grade inflammation is considered as one of the most important underlying pathophysiological mechanisms of this syndrome [85–87]. In addition, mast cells lie in proximity to nerve endings in the gut wall, a key position for the crosstalk between the immune and nervous systems of the gut [88]. Moreover, activated mast cells in proximity of nerve endings correlate significantly with the severity and frequency of abdominal pain in IBS patients [85, 88]. Interestingly, miRNAs regulate both immune and nervous systems along with control signal exchange in neuro-immune interactions involved in pain pathways [89]. For instance, miR-490-5p expression promotes mast cell proliferation and resistance to apoptosis, probably via multiple targets, including the tryptase/PAR-2 signal path [90]. Moreover, the downregulation of miR-125b and miR-16 correlates with increased mast-cell counts in the jejunum biopsies of IBS-D patients [19].

A further study carried out on an established IBS animal model provided evidence that miR-181c-5p overexpression determined IL1A downregulation exerting anti-inflammatory effects in IBS [91].

An altered interaction between sexual hormone production and immune response was reported in IBS patients [92–94]. Recently, three different miRNAs, namely miR-145, miR-148-5p, and miR-592, involved in these interactions were found dysregulated in IBS patients. In particular, miR-145 and miR-592 expressions were decreased in IBS-C and IBS-D patients, respectively, while miR-148-5p levels were higher in IBS-D patients compared to control subjects [95]. A recent study highlighted the involvement of eosinophils in IBS-D, via peripheral CRF. The same study also reported the corticotropin-releasing hormone receptor 1 gene (CRHR1) downregulation and the up-regulation of the membrane protein SNAP23 related to vascular transport in IBS-D [96]. Numerous lines of evidence suggest a positive effect of HDAC inhibition at the central level and an immune modulation mediated by microglial cells [97–100]. In particular, Jaworska and colleagues showed that these effects are induced by SB, which also promoted central neurogenesis in rat pups subjected to central ischemic insult [100]. Furthermore, rats perfused with SB into the cecum showed a modified enteric neuronal plasticity favouring the reinforcement of the cholinergic system associated with an increased visceral hypersensitivity as described in the following paragraphs [48]. Since SB can cross blood–brain-barrier due to its low-molecular weight, its effect at a central level has been thoroughly investigated. A recent study showed how dietary intervention could suppress microglia activation in the CNS after an excessive alcohol intake mainly through suppressing neuroinflammation [101]. The emerging clue is that SB could restore gut dysbiosis, upgrading beneficial bacteria and their metabolites. This improves the intestinal microenvironment, thereby exerting a protective effect on the intestinal mucosal barrier and nervous system. However, further studies are warranted to clarify SB-induced gut-brain neuroplastic changes and to define the underlying cellular and molecular mechanisms, particularly those related to HDAC-mediated gene expression. Lately, several studies delved more into these mechanisms showing a protective role of SB on different in vivo models of ischemic stroke inhibiting the expression of proinflammatory cytokines TNF-α and nitric oxide synthase-1, while upregulating IL-10 [102, 103]. Still at a central level, akin results showed improved neuromotor capabilities of mice models of Parkinson’s Disease treated with SB, linking this effect to an inhibition of the TLR4/MyD88/NF-B inflammatory pathway, as well as to a beneficial effect on intestinal barrier elicited by a reduction of dysbiosis [102]. Although related to a different experimental model, these results are in contrast with a possible detrimental activity of SB described previously in different experimental models. Yet, a clear causal link clarifying SB-induced epigenetic changes in regulating neuro-immune interactions at a central level and IBS is still missing.

### Serotonin signalling

The intestinal tract represents the major site of serotonin (5-hydroxytryptamine, 5-HT) production. The enterochromaffin cells produce up to 95% of the total 5-HT in the human body. 5-HT regulates intestinal motility, peristalsis, and contributes to several physiological functions such as neurogenesis and bone mass accrual [104]. The serotoninergic system comprises 15 different receptors of which 5HT3 and 5HT4 are the main targets for therapeutic strategies currently applied in IBS-D and IBS-C treatment, respectively. Indeed, experimental evidence showed a dysregulation of 5HT system in at least a subgroup of IBS, and more recently the influence of the gut microbiota to host serotoninergic system has become the target of research [105, 106]. This also includes a novel scenario where resident bacteria produce 5HT to activate host’s enterochromaffin cells [107].

From a biochemical standpoint, 5HT is also responsible for the covalent binding of proteins mediated by transglutaminases (TG), a process defined as serotonylation [108] which occurs at trimethylated histone 4 (H4K3me3). A recent milestone study described a causal link between serotonylation of H4K3me3 via TG2 and euchromatin levels, setting off this process as an additional epigenetic modification mostly occurring in the brain and colon [109]. Interestingly, H4K3me3 levels were associated with intestinal permeability and a decrease of *Lactobacillus plantarum* L168 in the intestinal tract of a *Drosophila Melanogaster* model [110].

Coming to non-coding RNA dysregulation mediated by genetic variants, Kapeller and colleagues showed that the 5HT3 variant c.*76G>A, located in the 3’UTR regulatory region of the E subunit (5HT3E), was associated with female IBS-D. The authors demonstrated that this variant causes the loss of the binding site of miR-510 inducing an increased expression of 5HT3 [111]. Another study reported a significant downregulation of miR16 and miR-103 in jejunum biopsies of IBS-D patients compared to controls [112]. Decreased levels of these miRNAs correlated with IBS symptom severity through 5-HT4 mRNA targeting [112]. 5-HT recaption and metabolism by enterocytes are mediated by SERT and monoamine oxidase activity, respectively [109, 113]. A growing consensus indicates an altered SERT expression in IBS patients [114, 115]. Interestingly, numerous studies described the involvement of different miRNAs in the regulation of SERT gene expression. miR-16, previously mentioned as a 5HT3 inhibitor and associated with high mast cell count, was associated with reduced SERT expression at a central level in mice raphe nuclei [116]. These results were further confirmed by Moya and colleagues which, additionally, observed a repressive activity on SERT exerted by miR-15a [117]. Furthermore, miR-200a upregulation was associated with a downregulation of both CNR1 and SERT in a rat model of IBS-D [79]. Finally, miR-24 was increased in the colonic mucosa of IBS patients compared to healthy subjects as well as in mice models induced by TNBS, together with a decrease of SERT levels [114]. In addition, the above-mentioned miR-29a can modulate the translation of the HTR7 protein, a G-protein-coupling receptor of 5-HT. Specifically, the overexpression of miR-29a, observed in the colon tissue of IBS patients and in WAS model, reduces the HTR7 expression, enhancing visceral hyperalgesia [118].

### Dysregulation of HPA axis and visceral hypersensitivity

Intestinal over-responsiveness to noxious stimuli is a crucial feature in IBS, resulting from several molecular mechanisms promoting peripheral and central sensitization [119]. Recent findings showed a higher colorectal contraction in response to corticotropin-releasing factor (CRF), and an altered activity at a central level upon colorectal distension (CRD), confirming the contribution of the HPA axis in IBS visceral pain [120]. In addition, it has been observed an increased amount of CRF in the cytoplasmic granules of jejunal eosinophils in IBS-D patients positively correlated with IBS clinical severity, chronic stress, and depression [96]. Importantly, the evidence demonstrated that subjects reporting EAEs and/or psychiatric disorders showing an increased activity of HPA-axis [3, 7] are prone to develop visceral hypersensitivity and IBS in adulthood [6, 121]. Notably, HDAC inhibitor MS-275 showed a beneficial effect on the anxiety-prone mice model, proposing H3 acetylation as a possible biomarker for the development of anxiety therapies [122].

From the scientific databases consulted in the present work we were not able to retrieve any data on epigenetic variation in visceral hypersensitivity associated with IBS with no central comorbidities, which has been extensively described by Mahurkar-Joshi and Chang [123]. Notably, two independent animal studies detected an increased visceromotor response (VMR) to CRD associated with HDAC inhibition induced by SB treatment, further suggesting SB as a contributor to IBS symptoms [124, 125].

**Fig. 2:**
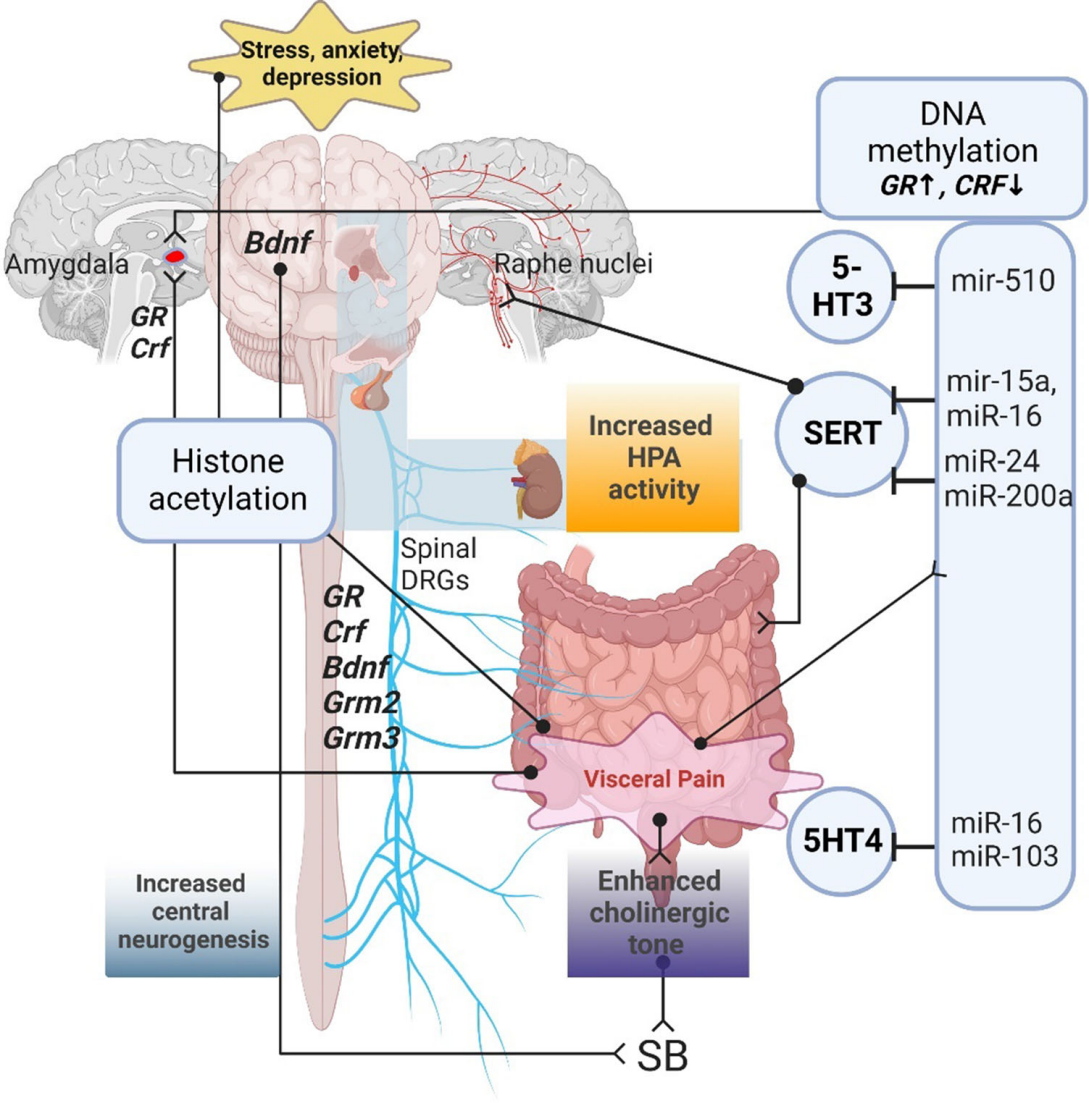
Epigenetic factors involved in brain-gut axis dysregulation. The scheme reports gene acetylation and miRNAs evoking visceral pain by interacting with key players of serotonergic (i.e., 5-HT3, 5-HT4, SERT), cholinergic, HPA response, and neurogenesis. *SERT* serotonin transporter, *SB* sodium butyrate, *GR* glucocorticoid receptor, *CRF* corticotropin-releasing factor, *BDNF* brain-derived neurotrophic factor, *GRM* glutamate receptor genes

Translational models demonstrated that stress or painful events in early life predisposed to chronic pain [126] and higher visceral hypersensitivity [127] via epigenetic regulation. Moloney and colleagues showed that in a rat model of maternal separation, visceral hypersensitivity was associated with a reduction of histone 4 lysine 12 (H4K12) acetylation in tissue samples from the spinal cord. Furthermore, inhibition of HDAC by treating animals with suberoylanilide hydroxamic acid (SAHA) reduced the evacuation frequency and pain sensation triggered by CRD [128]. In line with this, histone acetylation as well as DNA methylation was increased in male rats subjected to WAS and corticosterone treatment [21]. Moreover, increased signalling of brain-derived neurotrophic factor (BDNF) and glucocorticoid gene expression detected in animal models of EAEs are mediated by epigenetic factors [129, 130]. Notably, BDNF was increased in the colonic biopsies of patients with IBS [131, 132] and its concentration correlated with IBS symptom severity [131] and visceral hypersensitivity [132]. BDNF upregulation derived by H3 acetylation and HDAC1 inhibition was associated with enhanced VMR only in female rats subjected to heterotypic intermittent chronic prenatal stress [133]. This is also consistent with the altered regulation of BDNF observed in HPA-related disorders associated with early life stress [129, 134]. Interestingly, Lambert and colleagues extensively described a stress-dependent induction of glucocorticoid receptor (GR) mediated by BDNF, showing an exclusive, synergic activity of dexamethasone and BDNF in inducing an altered transcriptomic trait of central neurons [135]. As none of the functions of the detected mRNA profile was ascribable to BDNF nor GR activity taken alone, the authors hypothesized an epigenetic regulation exerted by BDNF on GR as a possible underlying mechanism. In line with this evidence, increased acetylation of lysine 9 on histone 3, (H3K9) was displayed at both GR and CRF promoter sites in a rat model of odour-induced stress showing enhanced VMR [136] (Fig. 2). In addition, in the amygdala of rats, a higher VMR induced by WAS was associated with an increased promoter methylation and reduced expression of GR. On the other side, CRF promoter methylation was decreased with a concomitant increase in CRF expression. In addition, visceral hypersensitivity was decreased upon treatment with the HDAC inhibitor TSA [137]. Hong and colleagues showed that rats subjected to WAS displayed an increase in DNA methylation of the glucocorticoid receptor (NR3C1) and CNR1 promoters and increased histone acetylation of transient receptor potential vanilloid type 1 (TRPV1) promoter [21]. As previously mentioned, IL-6 promotes H3K9 methylation in a GR-mediated way in colon epithelial cells and prevents TJs proteins expression, resulting in paracellular permeability increment and visceral hyperalgesia increase [71].

Experimental models of post-infectious IBS showed that increased vasoactive intestinal peptide (VIP) levels in the *muscolaris externa* mediated an upregulation of the voltage-gated calcium receptor 1.2b subunit α1C1b through H3K9. This could allegedly increase intracellular calcium flux resulting in enhanced smooth muscle contractions [124].

Rats with increased VMR induced by forced swim showed a decreased acetylation (H3K9, H3K18) and expression levels of glutamate receptor genes *Grm 2* and *Grm3* [138]. Furthermore, the same authors detected a lower H3K9 acetylation of *Grm 2* associated with an increased VMR derived by estradiol substitutive supplementation in ovariectomized female rats [139]. These data indicate gonadal hormones as possible contributors to the development of visceral hypersensitivity induced by early-life stress events. Notably, the VMR of both ovariectomized and forced-swim models was reduced by restored levels of GR induced by HDAC inhibition [138, 139]. In line with these findings, the glutamatergic system might also be dampened by a reduction of glutamate decarboxylase described in pain disorders as well as by the inhibition of the GLUL gene by miR-29a described in IBS patients [74].

In a rat model of IBS-D and in human colonic biopsies of patients with IBS-D, elevated levels of miR-200a have been shown. MiR-200a seems to be involved in increasing visceral hypersensitivity by acting on CNR1 and SERT [79]. Moreover, the miR-495 was poorly expressed in a mice IBS-D model, whereas PKIB, the miR-495 target gene was upregulated. On the other hand, overexpression of miR-495 and the consequent suppression of PKIB can inhibit the PI3K/AKT signalling pathway and decreases visceral sensitivity [140]. An additional study reported that a decreased miR-325-5p expression correlates with CCL2 upregulation in a rat model of chronic visceral pain [141]. The involvement of CCL2 in inflammatory and neurodegenerative diseases and functional dyspepsia [141–143] point to the involvement of the miR-325-5p/CCL2 signalling in chronic visceral pain in IBS patients and other patients with the gastrointestinal disorder as well.

## Conclusions

Increasing experimental evidence suggests the involvement of epigenetic regulation in IBS pathophysiology. Residing intestinal microbiome could represent a hub between the different mechanisms involved in IBS, whilst epigenetic factors should be reckoned as contributors for implementing these mechanisms in the host. Moreover, recent findings describe crypt enterocytes as key players in host-microbiome interaction mediated by both histone modifications [49, 53] and miRNA signalling [23]. The indirect activity of microbial metabolites such as that exerted by SB-induced HDAC inhibition represents a complex multifactorial regulation of adaptive immunity, intestinal motility, the permeability of gut barrier, and abdominal pain. At the host interface, extra-cellular miRNA produced by intestinal epithelial and stem cells contribute to the equilibrium between residing microbial species [65]. With regard to ncRNA, many miRNAs have been identified as implicated in the pathophysiology of IBS. Among these, the miR-19 family implicated in both visceral hypersensitivity and augmented intestinal permeability could represent key biomarkers in IBS diagnosis and treatment, particularly for IBS-D patients. However, additional studies are needed to confirm the role of this miRNA family and identify enzymes and signalling paths involved in IBS complexity.

An important limitation in IBS preclinical research pertains to the complexity and heterogeneity of this disease, which pose a hurdle to the development of a reliable and valid experimental model. For this reason, there is no animal model of IBS mimicking all the symptoms typical of this condition. Nowadays, there are only models imitating some aspects of IBS. Neonatal maternal separation, water avoidance, and wrap restraint stress are useful to study the mechanisms underlying psychological stress, but they cannot be considered a model of IBS. Several studies on epigenetics in IBS belong from animal models, representing a major limitation in the evaluation of the results.

Future studies should include a large cohort of well-characterized patients to decipher the molecular mechanisms, including epigenetic ones, underlying IBS pathophysiology and to identify new therapeutic options.

## Abbreviations

AHR: Aromatic hydrocarbon receptor
ARF4: ADP ribosylation factor 4
BDNF: Brain-derived neurotrophic factor
CNR1: Cannabinoid receptor 1
CRD: Colorectal distension
CRF: Corticotropin-releasing factor
DNMTs: DNA methyl transferase
DRG: Dorsal root ganglia
EAEs: Early-life adverse events
EGC: Enteric glial cells
EV: Extracellular vesicles
FSS: Forced swim stress
FODMAPs: Fermentable oligo- mono- di-saccharides and polyoils
GF: Germ free
GMR: Glutamate metabotropic receptor
GR: Glucocorticoid receptor
HATs: Histone acetyl transferase
HDACs: Histone deacetylases
HPA: Hypothalamic–pituitary–adrenal
IECs: Intestinal epithelial cells
IP: Intestinal permeability
lncRNA: Long non-coding RNA
miRNA: MicroRNA
MS: Maternal separation
NGF: Neuronal growth factor
RGS2: Regulator of G protein signalling 2
SAHA: Suberoylanilide hydroxamic acid
SB: Sodium butyrate
SCFAs: Short chain fatty acids
SERT: Serotonin transporter
TNBS: Trinitrobenzene sulfonic acid
TSA: Trichostatin
VIP: Vasoactive intestinal protein
VMR: Visceromotor response
WAS: Water avoidance stress
5HT: 5-Hydroxytryptamine serotonin
5HT_3_: Serotonin receptor 3
5HT_4_: Serotonin receptor 4
